# Molecular identification and genetic variations of forensically significant blow flies (Diptera: Calliphoridae) from Eastern India using DNA barcoding

**DOI:** 10.1371/journal.pone.0327039

**Published:** 2025-07-22

**Authors:** Oishik Kar, Arka Mukherjee, Koustav Mukherjee, Debdeep Pramanik, Atanu Naskar, Dhriti Banerjee

**Affiliations:** Diptera Section, Zoological Survey of India, Kolkata, West Bengal, India; Instituto Leonidas e Maria Deane / Fundacao Oswaldo Cruz, BRAZIL

## Abstract

Flies, especially those from the Calliphoridae family, play a crucial role in decomposition and are the first to colonize a cadaver. Firstly, accurate species identification is a prerequisite for entomological evidence-based calculation of postmortem interval (PMI). While morphological criteria for identifying the species of adult blow flies exist, there are either absent or inadequate keys for younger stages. In all phases of blow fly development, molecular identification offers a quick and accurate procedure. It is widely known that mitochondrial cytochrome oxidase subunit I has the capacity for molecular identification but is ineffective in certain species. This study was conducted to assess the effectiveness of the cytochrome oxidase 1 gene in the identification of seventeen different species of calliphorid flies involving four genera, *Calliphora*, *Chrysomya*, *Lucilia*, and *Hemipyrellia*. In West Bengal, 2,977 blow fly specimens were gathered from four distinct geo-climatic zones. COI barcodes were able to confirm morphological identification through low K2P intraspecific genetic divergences (0% to 1%) and moderate to high K2P interspecific genetic divergences (0.39% to 12.29%). The Neighbour-Joining (NJ) analysis demonstrated well-supported reciprocal monophyly among the species. The species grouping was in agreement with morphological and molecular identifications. The four delimitation methods, BIN, ASAP, PTP, and GMYC, used for species identification produced similar results and facilitated the proper identification of species. Therefore, it can be concluded that COI barcodes are a highly successful alternative for the molecular identification of blow flies, facilitating forensic cases and biodiversity research in India.

## Introduction

The Calliphoridae family of blow flies is one of the most researched families worldwide [1]. The forensic, medicinal, veterinary, and economic fields are all directly affected by the synanthropic habits of these blow flies [2–12]. Specifically, blow flies are among the first organisms to colonize corpses, indicating that they might act as a biological clock to determine the time of death and play a significant role in determining minimum post-mortem interval (minPMI) [5]. Forensic entomologists most frequently utilize immature blow fly stages like egg, larva, pupa, etc., retrieved from a crime scene to ascertain the postmortem interval (PMI) in death investigations [13,14]. Since several closely related species of blow flies have different developmental temperature thresholds and grow differently at the same temperature, proper identification of these species is essential for accurate PMI calculation [15–18]. Erroneous species determinations can lead to faulty and inaccurate estimates of the PMI and other conclusions [19]. The identification of species has traditionally been dependent upon morphological characteristics, requiring the knowledge of qualified and expert taxonomists. Unfortunately, this method is highly laborious and time-consuming, and it cannot accurately assess damaged specimens or distinguish between sibling species [20,21] mainly when accurate taxonomic keys are not available and specimens are poorly preserved [22]. In response to these constraints, molecular biology techniques have been added exponentially to morphological identification to provide quick and accurate species identification. The molecular identification procedure also simplifies differentiating closely related cryptic species with similar adult or immature life-stage traits [23]. A forensic entomologist in these circumstances would greatly benefit from molecular identification of the available samples to correctly identify entomological evidence [24].

The mitochondrial cytochrome oxidase I (COI) gene, which is a potent tool for mt-DNA-based analysis, can help address the problems of traditional taxonomy even in cases when a suitable specimen for morphological identification cannot be received [25–29]. Blow fly species have been identified using sequence data from both nuclear and mitochondrial loci [1,24,30–57]. The main reasons why mitochondrial loci are usually chosen over nuclear loci are that they are present in multiple copies, which makes them helpful in amplifying damaged samples, they evolve much more quickly than nuclear DNA, which makes them helpful in identifying closely related sister species, they do not undergo recombination, which reduces intraspecies variation, and they are readily available as universal primers for an extensive range of blow fly species [58]. Nevertheless, some studies on molecular identification of calliphorid genera like *Lucilia* have revealed different results, where the nuclear loci (ITS2) was more efficient than cytochrome oxidase I (COI) or other markers were used instead of COI [36,43,48,51].

Previously, studies on molecular identification and characterization of blow fly species from northern, southern, and central parts of India have been conducted [24,29,44,46,56,59–64]. All of the research indicated the robustness of the cytochrome oxidase I (COI) gene in the molecular identification of different species of blow flies. However, COI failed to discriminate between closely related species of *C. megacephala* and *C. chani* [24]. Besides COI, 16s rRNA was successfully used as a molecular approach to identify blow fly species [44].

Located between the Bay of Bengal to the south and the Himalayas to the north, the state of West Bengal is situated in the eastern part of India. The geographical distribution of climatic zones in the region includes: the flatlands of the Gangetic plain (which includes Howrah, Hooghly and Kolkata), the mountainous region (which consists of Darjeeling, Kalimpong and Jalpaiguri), semi-desert or arid region (including Bankura, Birbhum, and parts of Murshidabad) and the marine coastal belt area (including South 24 Paraganas and Sagar Islands and eastern part of Midnapore) [65]. With such diverse geo-climatic zones, the diversity of blow flies throughout the state would vary considerably.

This study aim to (1) identify the species of Calliphoridae important for forensic studies in east India, (2) produce mitochondrial COI sequencing data for the accurate and proper molecular DNA-based identification of blow flies from east India, (3) make available sequence barcode data in GenBank and BOLD for future genetic studies, and (4) it will aid in understanding the complete scenario of the distribution and diversity of blow flies in this region, ultimately enriching forensic entomological studies.

## Materials and methods

### Sample collection and identification

**Fig 1:**
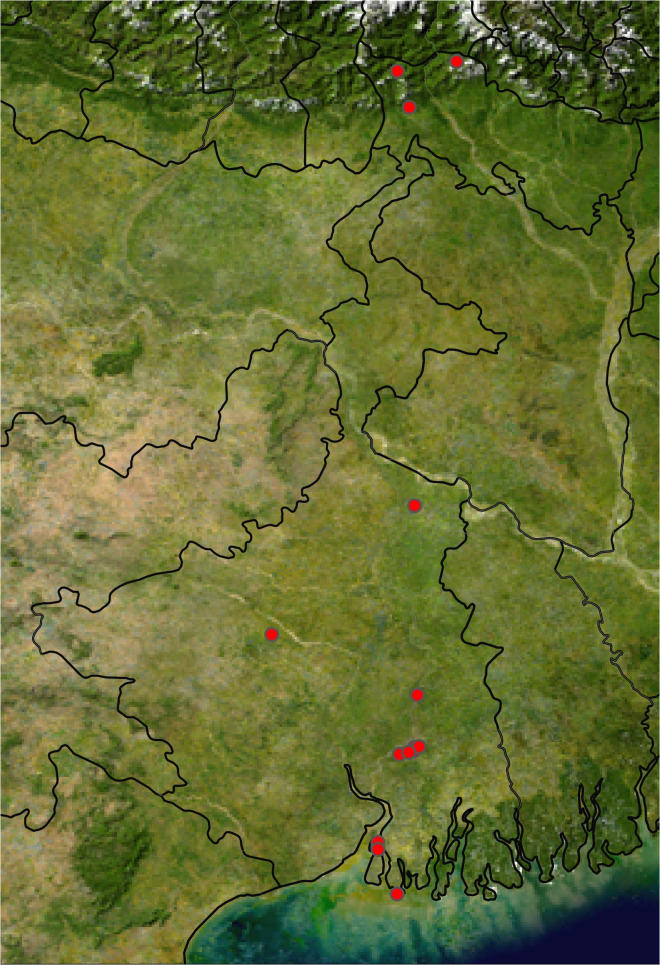
Map representing the sampling localities of different blow fly species from four different geo-climatic regions of West Bengal, India.

Adult blow fly specimens were caught using sweep nets and bait traps [66] from four geo-climatic regions [65] of West Bengal (Table 1) from January 2023 to December 2023 encompassing pre-monsoon (March, April, May, and June), monsoon (July, August, September, and October), and post-monsoon (November, December, January, and February) [67]. The permit for working in the mentioned field sites was approved by the Directorate of Forests, Government of West Bengal, vide Memo No.: 2059/WL/4R-4 (Pt-II)/2021. Flies were collected for two hours in the morning (9.00 A.M.-11.00 A.M.) per site. Each collection site was visited once in each of the aforementioned seasons (pre-monsoon, monsoon, and post-monsoon). The traps were baited with putrefied goat flesh, chicken liver, and decaying fish remains, and net sweeping was done in and around the traps. Subsequently, fly specimens were transferred to Falcon tubes (DNase-free) and stored in high-grade ethyl alcohol (70%). Specimens were morphologically identified up to the species level using a stereomicroscope and taxonomic keys [68–70]. A Leica stereo-iso microscope M205A, coupled with a Leica DFC 500 camera and Leica Application Suite LAS v3.6 software, was implemented for snapping photographs of the fly specimens. Valid species names were assigned according to the Systema Dipterorum v5.0 [71]. 52 specimens representing the 21 species collected in this region were selected for molecular studies (Table 1). One or two legs were collected from individual species specimens. The remaining voucher specimens were kept and stored in the insectarium at the National Zoological Collection (NZC), Diptera Section, Zoological Surveys of India (HQ), Kolkata. The map (Fig 1) was created using an online software named SimpleMappr (http://www.simplemappr.net) [72], following the input of the geological coordinates of the study locations with the help of a Garmin GPS device.

**Table 1:** List of species used in the DNA-Barcode analyses. Collected specimens in West Bengal include details of locality and voucher numbers.

Name of the species	Voucher Number	Locality	Coordinates	GenBank Accession Number	Sequences accessed from GenBank
*Chrysomya rufifacies* (Macquart, 1844)	DIP_54	Sapkhali, Sagar Island	21.862 N, 88.129 E	OL414948	MT502111 (Nagpur, India), MK038735 (Tamil Nadu, India), MK353338 (Jammu, India), KT894979 (Thailand), PP581363 (Thailand), KT894981 (Thailand)
*Chrysomya rufifacies* (Macquart, 1844)	DIP_62	Sapkhali, Sagar Island	21.862 N, 88.129 E	OL660767	
*Chrysomya rufifacies* (Macquart, 1844)	DIP_63	New Alipore, Kolkata	22.509 N, 88.333 E	PP339414	
*Chrysomya rufifacies* (Macquart, 1844)	DIP_64	Rishop, Kalimpong	27.112 N, 88.653 E	PP318763	
*Chrysomya rufifacies* (Macquart, 1844)	OK_24	Sonamukhi, Bankura	23.294 N, 87.420 E	PQ146924	
*Chrysomya megacephala* (Fabricius, 1794)	DIP_53	Sapkhali, Sagar Island	21.862 N, 88.129 E	PP339752	MZ461937 (Tamil Nadu, India), KX893341 (Kerala, India), KU543646 (USA), KT894991 (Thailand), LC549067 (Malaysia), MZ769396 (Malaysia), JQ246662 (Brazil)
*Chrysomya megacephala* (Fabricius, 1794)	DIP_61	Sapkhali, Sagar Island	21.862 N, 88.129 E	OL673808	
*Chrysomya megacephala* (Fabricius, 1794)	DIP_70	Ghoramara Island, South 24 Parganas	21.911 N, 88.128 E	ON197878	
*Chrysomya megacephala* (Fabricius, 1794)	DIP_71	Bakkhali, South 24 Parganas	21.565 N, 88.256 E	ON248447	
*Chrysomya megacephala* (Fabricius, 1794)	DIP_74	Rishop, Kalimpong	27.112 N, 88.653 E	PP330089	
*Chrysomya megacephala* (Fabricius, 1794)	OK_25	Sonamukhi, Bankura	23.294 N, 87.420 E	PQ146958	
*Chrysomya megacephala* (Fabricius, 1794)	OK_26	New Alipore, Kolkata	22.509 N, 88.333 E	PQ146973	
*Chrysomya megacephala* (Fabricius, 1794)	OK_27	Dhapa, Kolkata	22.547 N, 88.402 E	PQ146975	
*Chrysomya bezziana* Villeneuve, 1914	DIP_72	Dhapa, Kolkata	22.547 N, 88.402 E	PP330056	MK167359 (Jammu, India), PP581388 (Thailand), PP581387 (Thailand), AF295548 (Malaysia), JQ246660 (Malaysia)
*Chrysomya bezziana* Villeneuve, 1914	OK_28	Sapkhali, Sagar Island	21.862 N, 88.129 E	PQ147036	
*Chrysomya bezziana* Villeneuve, 1914	OK_29	Ruhia, Murshidabad	24.153 N, 88.373 E	PQ147040	
*Chrysomya bezziana* Villeneuve, 1914	DIP_73	Gangatala, Hooghly	22.891 N, 88.390 E	PP330058	
*Chrysomya nigripes* Aubertin, 1932	OK_30	Sonamukhi, Bankura	23.294 N, 87.420 E	PQ148151	KX893349 (Kerala, India), OR044072 (China), KR921621 (Thailand)
*Chrysomya nigripes* Aubertin, 1932	OK_31	Gangatala, Hooghly	22.891 N, 88.390 E	PQ148156	
*Chrysomya pinguis* (Walker, 1858)	OK_33	Sapkhali, Sagar Island	21.862 N, 88.129 E	PQ148319	KX893350 (Himachal Pradesh, India), FJ195381 (Nepal), KY001888 (China), KT894995 (Thailand)
*Chrysomya pinguis* (Walker, 1858)	OK_32	New Alipore, Kolkata	22.509 N, 88.333 E	PQ148309	
*Chrysomya pinguis* (Walker, 1858)	OK_34	Sonamukhi, Bankura	23.294 N, 87.420 E	PQ151614	
*Chrysomya defixa* (Walker, 1856)	OK_40	Rishop, Kalimpong	27.112 N, 88.653 E	PQ151656	LC549104 (Malaysia), LC549090 (Malaysia)
*Chrysomya defixa* (Walker, 1856)	OK_41	Sapkhali, Sagar Island	21.862 N, 88.129 E	PQ151661	
*Chrysomya albiceps* (Wiedemann, 1819)	N/A	N/A	N/A	N/A	MF695707 (Brazil), MF695684 (Brazil)
*Chrysomya chani* Kurahashi, 1979	N/A	N/A	N/A	N/A	MW600494 (Kerala, India), KT894988 (Thailand), KT894989 (Thailand), KR921608 (Thailand), KR921609 (Thailand), FJ195377 (Vietnam), PP581391 (Thailand)
*Chrysomya thanomthini* Kurahashi & Tumrasvin, 1977	N/A	N/A	N/A	N/A	KT894997 (Thailand), FJ195386 (Vietnam)
*Chrysomya villeneuvi* Patton, 1922	N/A	N/A	N/A	N/A	KT894983 (Thailand), KR921638 (Thailand), PP581379 (Thailand)
*Hemipyrellia ligurriens* (Wiedemann, 1830)	DIP_75	Rishop, Kalimpong	27.112 N, 88.653 E	OL415087	MN831480 (Kerala, India), EU880206 (Republic of Korea), KY031875 (China), LC549069 (Japan), FJ614822 (China), JN014895 (Malaysia)
*Hemipyrellia ligurriens* (Wiedemann, 1830)	DIP_76	Sapkhali, Sagar Island	21.862 N, 88.129 E	ON171479	
*Hemipyrellia ligurriens* (Wiedemann, 1830)	DIP_77	Dhapa, Kolkata	22.547 N, 88.402 E	PP330125	
*Hemipyrellia ligurriens* (Wiedemann, 1830)	DIP_78	Gauripur, South 24 Parganas	22.496 N, 88.270 E	PP330209	
*Hemipyrellia pulchra* (Wiedemann, 1830)	DIP_90	Dhapa, Kolkata	22.547 N, 88.402 E	PP330738	KR921680 (Thailand)
*Hemipyrellia pulchra* (Wiedemann, 1830)	DIP_91	Ruhia, Murshidabad	24.153 N, 88.373 E	PP338114	
*Calliphora vomitoria* (Linnaeus, 1758)	DIP_79	Rishop, Kalimpong	27.112 N, 88.653 E	PP330225	PP267941 (Turkey), KF918995 (Belgium), KY001919 (China), MG969488 (Republic of Korea)
*Calliphora vomitoria* (Linnaeus, 1758)	DIP_80	Happy Valley, Darjeeling	27.048 N, 88.258 E	PP330226	
*Calliphora vicina* Robineau-Desvoidy, 1830	DIP_88	Sukna Forest, Darjeeling	26.807 N, 88.338 E	PP330399	KX893334 (Punjab, India), OP503181 (Chandigarh, India), KF918988 (Belgium), KF225220 (Spain)
*Calliphora vicina* Robineau-Desvoidy, 1830	DIP_89	Rishop, Kalimpong	27.112 N, 88.653 E	PP330413	
*Calliphora pattoni* Aubertin, 1931	OK_50	Rishop, Kalimpong	27.112 N, 88.653 E	PQ164419	DQ345095 (China)
*Calliphora pattoni* Aubertin, 1931	OK_51	Sukna Forest, Darjeeling	26.807 N, 88.338 E	PQ164426	
*Lucilia papuensis* Macquart, 1844	OK_5	Gariahat Market, Kolkata	22.519 N, 88.366 E	PP264290	KT895004 (Thailand), KR921652 (Thailand), KR921656 (Thailand)
*Lucilia papuensis* Macquart, 1844	OK_10	Sonamukhi, Bankura	23.294 N, 87.420 E	PP338117	
*Lucilia papuensis* Macquart, 1844	OK_12	Ruhia, Murshidabad	24.153 N, 88.373 E	PP338120	
*Lucilia porphyrina* (Walker, 1856)	OK_11	Dhapa, Kolkata	22.547 N, 88.402 E	PP231813	KX893336 (Punjab, India), KX893338 (Punjab, India), MF694302 (China), KM497299 (China), KX096343 (Thailand), KT895002 (Thailand)
*Lucilia porphyrina* (Walker, 1856)	OK_3	Dhapa, Kolkata	22.547 N, 88.402 E	PP195945	
*Lucilia porphyrina* (Walker, 1856)	OK_15	Sonamukhi, Bankura	23.294 N, 87.420 E	PP338124	
*Lucilia porphyrina* (Walker, 1856)	OK_16	Ruhia, Murshidabad	24.153 N, 88.373 E	PP339765	
*Lucilia sericata* (Meigen, 1826)	OK_17	Sonamukhi, Bankura	23.294 N, 87.420 E	PP338156	MT483868 (China), PP267948 (Turkey), KU543649 (USA)
*Lucilia sericata* (Meigen, 1826)	OK_18	New Alipore, Kolkata	22.509 N, 88.333 E	PP338185	
*Lucilia ampullacea* Villeneuve, 1922	OK_19	Sukna Forest, Darjeeling	26.807 N, 88.338 E	PP338189	MN609625 (Republic of Korea), JX295666 (Belgium), MZ626876 (Sweden), LT963485 (UK)
*Lucilia ampullacea* Villeneuve, 1922	OK_20	Rishop, Kalimpong	27.112 N, 88.653 E	PP338246	
*Lucilia ampullacea* Villeneuve, 1922	OK_21	Happy Valley, Darjeeling	27.048 N, 88.258 E	PP338255	
*Lucilia illustris* (Meigen, 1826)	OK_22	Park Circus, Kolkata	22.542 N, 88.372 E	PP338257	JX295721 (Belgium), KC510144 (South Korea), KM570007 (Canada), FJ614827 (China)
*Lucilia illustris* (Meigen, 1826)	OK_23	Happy Valley, Darjeeling	27.048 N, 88.258 E	PP338264	
*Lucilia cuprina* (Wiedemann, 1830)	OK_42	Park Circus, Kolkata	22.542 N, 88.372 E	PQ152239	KR921645 (Thailand), KR921648 (Thailand), OQ519770 (China), KY797311 (Costa Rica), KC568275 (Colombia), MT483882 (Peru), MF536031 (Lebanon), KJ129383 (China)
*Lucilia cuprina* (Wiedemann, 1830)	OK_43	Sonamukhi, Bankura	23.294 N, 87.420 E	PQ152299	

(N/A- Not Applicable).

### DNA extraction, Polymerase Chain Reaction (PCR) amplification of COI gene, and DNA Sequencing

DNA was extracted using the QIAmp DNA extraction kit (QIAGEN, Germany) from one or two crushed leg tissues of the fly specimen [57]. The manufacturer’s protocol was followed to complete the procedure. Extracted DNA was quantified using a Qubit Fluorometer (Life Technologies, USA) and stored at −20°C for further examination. Amplification of about 700 base pairs from the mitochondrial COI gene 5’ end was performed using roughly 20 ng of genomic DNA employing primers forward LCO-1490 and reverse HCO-2198 (R) [73]. PCR was carried out according to [74]. To verify the size of the amplicon, the amplified products were observed on a 1% agarose gel, dyed with SYBR@safe DNA gel dye, and captured on an Invitrogen-safe gel imager.

The PCR-amplified products were purified with the help of the QIAquick Gel Extraction Kit (Qiagen, Germany) following the manufacturer’s instructions. About 15 ng of the purified PCR products were employed for cycle sequencing. Using both forward and reverse PCR primers, the BigDye®Termi-nator ver. 3.1Cycle Sequencing Kit (Applied Biosystems, Inc.) was utilized to perform cycle sequencing on an ABI thermal cycler with the following parameters: 96°C for 1 min, then 25 cycles of 96°C for 10 s, 50°C for 5 s, and final extension at 60°C for 1 min 15 s. After cycle sequencing, the products were cleaned with the BigDye X-terminator kit (Applied Biosystems Inc.) and placed into an ABI 3730 capillary Genetic analyzer at the Zoological Survey of India sequencing laboratory [75,76].

### Sequence analyses, data deposition, and dataset formation

Analyses were performed using publicly accessible COI sequences of other similar blow fly species obtained from the NCBI GenBank database. 138 blow fly COI sequences, spanning 21 species across 4 genera, were examined. 52 of those 138 sequences were produced for this study, representing the 4 genera and 17 species. The other 86 COI sequences from different parts of India and the world, and the out-group *Hydrotaea hennigi* (ON220743, India), a Muscidae [24] were acquired from GenBank (Table 1). The dataset was formed and initially aligned using the ClustalW algorithm in the MEGAX software [77]. To avoid incongruent outcomes, the dataset was made to be 616 base pairs long [78]. MEGAX software was used for forward and reverse chromatogram analysis of sequences produced from our collected species, along with sequence editing, which involved trimming both ends to remove any confusing bases and messy regions, followed by thorough annotation based on each sample’s forward and reverse sequences [79]. All our sequences were matched to identical reported sequences in the NCBI database using the BLAST (Basic Local Alignment Search Tool) (https://blast.ncbi.nlm.nih.gov) algorithm [80]. The ORF finder of NCBI (https://www.ncbi.nlm.nih.gov/orffinder/) was used to study the accurate amino acid codes devoid of any stop codon or insertions and deletions [79]. After that our sequences were uploaded to the NCBI GenBank database (https://submit.ncbi.nlm.nih.gov/) and BOLD systems (https://boldsystems.org), which generated unique accession numbers and BINs. The sequences in BOLD systems were uploaded under the project name “CALLIPHORIDAE FROM EASTERN INDIA” following their procedure.

### Genetic divergence and cluster analysis

The interspecific and intraspecific genetic divergences were estimated with the help of MEGAX involving Kimura-2-Parameter (K2P). The best-fit nucleotide substitution model was determined using JModelTest v2.1.10 [81] through the CIPRES server [82] and concerning the lowest AIC (Akaike Information Criterion) score of – 2764.8285 [79,83]. The best-fit nucleotide substitution model determined was the General time reversible model across lineages along with gamma and invariant (GTR + I + G) (NST = 6). A Neighbour-Joining (NJ) tree utilizing the Kimura-2-Parameter (K2P) was constructed to represent the divergence between the blow fly species [57]. The NJ tree was made in MEGAX, where the bootstrap consensus tree was inferred from 1000 replicates. The maximum likelihood (ML) tree dataset was created and examined in RAxML using the CIPRES website [82]. The Bayesian (BA) tree was constructed in Mr. Bayes v3.2.7a with nst = 6 for the (GTR + I + G) model and metropolis-coupled Markov Chain Monte Carlo (MCMC), which ran for 1,000,000 generations with 25% burn-in, conserving trees every 100 generations, to test the reciprocal monophyletic criteria for species delimitation. The FigTree v1.4.4 program (http://tree.bio.ed.ac.uk/software/fgtree/) and iTOL v6 tool (https://itol.embl.de/) were used to modify and construct trees from the produced files. As a result, the sequence divergence between the specimens was visually represented. DnaSP v5.10 [84] was used to estimate the haplotype diversity and the number of haplotypes.

### Species delimitation analyses

Additionally, assessments of species delimitation were conducted using single Poisson Tree Processes (PTP) [85], Generalised Mixed Yule Coalescent (GMYC) [86], Barcode Index Numbers (BINs) system [55,87], and Assemble Species by Automatic Partitioning (ASAP) [88]. The PTP analysis was done on the PTP web server (https://species.h-its.org/ptp/). The ASAP technique was performed online (https://bioinfo.mnhn.fr/abi/public/asap/). Kimura (K80) ts/tv, was used in ASAP for assessment. The GMYC analysis was performed on a web server version (https://species.h-its.org/gmyc/). All four delimitation methods were employed to determine the number of molecular operational taxonomic units (MOTUs). Every method produced a putative group of MOTUs. The following two categories were implemented to classify the produced MOTUs to verify their congruence: 1) match (both approaches identified a MOTU) and 2) no match (none of the approaches identified a MOTU) [57].

## Results

### Collection and morphological identification of specimens

**Fig 2:**
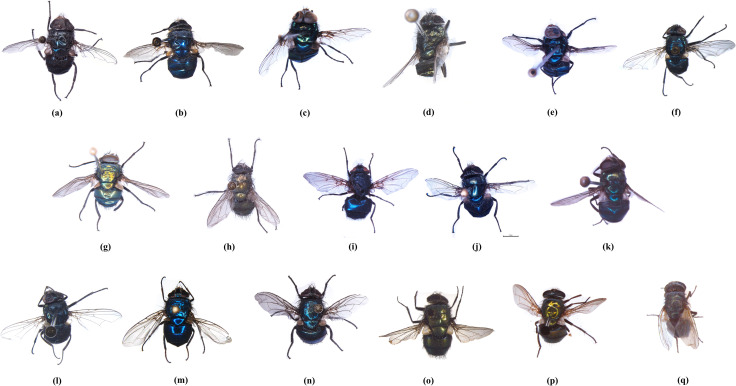
Images of species collected and barcoded for this study. (a) *Chrysomya megacephala* (Fabricius, 1794), (b) *Chrysomya bezziana* Villeneuve, 1914, (c) *Chrysomya rufifacies* (Macquart, 1844), (d) *Chrysomya nigripes* Aubertin, 1932, (e) *Chrysomya defixa* (Walker, 1856), (f) *Chrysomya pinguis* (Walker, 1858), (g) *Hemipyrellia ligurriens* (Wiedemann, 1830), (h) *Hemipyrellia pulchra* (Wiedemann, 1830), (i) *Calliphora vomitoria* (Linnaeus, 1758), (j) *Calliphora pattoni* Aubertin, 1931, (k) *Calliphora vicina* Robineau-Desvoidy, 1830, (l) *Lucilia ampullacea* Villeneuve, 1922, (m) *Lucilia porphyrina* (Walker, 1856), (n) *Lucilia papuensis* Macquart, 1844, (o) *Lucilia illustris* (Meigen, 1826), (p) *Lucilia cuprina* (Wiedemann, 1830), and (q) *Lucilia sericata* (Meigen, 1826).

A total of 2977 flies were collected from the four geo-climatic regions of West Bengal. These underwent morphological identification, resulting in the classification of eighteen species under four genera (Fig 2).

In general, collection in each season from four different geo-climatic zones revealed distinct results (Table 2). In all four regions, *Chrysomya megacephala* was the most prevalent species, accounting for 43.06% of the total, followed by *Chrysomya rufifacies*, which accounted for 23.41%.

**Table 2:** Abundance of blow flies from four different geo-climatic zones of West Bengal in different seasons.

		Study sites											
		Arid region			Coastal region			Gangetic plains			Hilly regions		
Forensic dipteran species	Total	PRM	MN	PST	PRM	MN	PST	PRM	MN	PST	PRM	MN	PST
*1. C. vomitoria*	57	0	0	0	0	0	0	0	0	0	26	23	8
*2. C. pattoni*	21	0	0	0	0	0	0	0	0	0	10	7	4
*3. C. vicina*	32	0	0	0	0	0	2	0	0	0	15	9	6
*4. C. bezziana*	109	18	2	0	22	3	0	37	18	9	0	0	0
*5. C. megacephala*	1282	101	98	53	180	107	69	255	238	62	75	43	11
*6. C. rufifacies*	697	75	40	22	101	50	13	155	132	48	42	13	6
*7. C. nigripes*	99	14	5	9	17	6	5	19	10	4	0	0	0
*8. C. pinguis*	47	0	0	0	12	9	1	10	3	1	6	5	0
*9. C. defixa*	9	0	0	0	3	0	0	2	0	0	4	0	0
*10. H. ligurriens*	207	20	34	0	27	29	2	49	20	11	13	2	0
*11. H. pulchra*	98	12	10	2	21	12	0	29	11	1	0	0	0
*12. L. ampullacea*	68	0	0	2	5	3	0	15	7	2	25	6	3
*13. L. cuprina*	71	10	6	3	20	6	4	5	5	2	5	3	1
*14. L. illustris*	43	0	1	2	11	5	1	17	3	3	0	0	0
*15. L. papuensis*	35	5	5	0	3	2	0	10	5	0	3	1	1
*16. L. porphyrina*	60	7	3	0	11	4	0	15	3	2	3	2	0
*17. L. sericata*	42	5	3	2	7	2	1	9	1	0	3	5	4
Total	2977												

(PRM- Pre-monsoon, MN- Monsoon, and PST- Post-monsoon).

### DNA sequences and barcode-based identification

The aligned dataset included 616 base pairs from the COI gene of 21 blowfly species belonging to the genera *Calliphora*, *Chrysomya*, *Hemipyrellia*, and *Lucilia*, which are significant for both forensic and medical reasons, respectively. The present study showed

The cytochrome oxidase I gene sequences had 189 conserved sites, 64 variable or polymorphic sites, 9 variable singleton sites, and 55 parsimony informative sites, excluding insertions, deletions, and other gap areas.

The 52 sequences from 17 species across four blow fly genera, identified by DNA barcoding, complemented morphological identifications. Following the BLASTN findings in GenBank, the flies belonging to the 17 species supported their morphological identifications, with sequence identities varying between 98% and 100% (Table 3). Similar results were found when we used the identification engine of the BOLD system. It displayed highest similarity and identification percentage (99.38%−100%) with the sequences of respective species in its database (Table 4).

**Table 3:** Identification of 17 collected blow fly species through NCBI BLASTN search tool.

Voucher Number	Maximum Identification	BLASTN in GenBank				
		Accession Number	Total Score	Query Coverage	E-value	Identity
DIP_54	*Chrysomya rufifacies*	KT894981	1249	99%	0.0	99.85%
DIP_62	*Chrysomya rufifacies*	KR921632	1090	100%	0.0	100%
DIP_63	*Chrysomya rufifacies*	KR921632	1184	100%	0.0	100%
DIP_64	*Chrysomya rufifacies*	KR921632	1157	100%	0.0	100%
OK_24	*Chrysomya rufifacies*	KT894981	1234	100%	0.0	100%
DIP_53	*Chrysomya megacephala*	MH778911	1160	100%	0.0	99.84%
DIP_61	*Chrysomya megacephala*	MH778920	1181	99%	0.0	99.69%
DIP_70	*Chrysomya megacephala*	MH778911	1134	100%	0.0	100%
DIP_71	*Chrysomya megacephala*	MH778911	870	98%	0.0	99.38%
DIP_74	*Chrysomya megacephala*	MZ461937	1096	100%	0.0	99.83%
OK_25	*Chrysomya megacephala*	MH778911	1146	100%	0.0	99.84%
OK_26	*Chrysomya megacephala*	MH778911	1151	100%	0.0	99.84%
OK_27	*Chrysomya megacephala*	PQ461657	1194	100%	0.0	100%
DIP_72	*Chrysomya bezziana*	JQ246660	1199	100%	0.0	100%
OK_28	*Chrysomya bezziana*	KR921597	1134	100%	0.0	100%
OK_29	*Chrysomya bezziana*	KR921597	1107	100%	0.0	100%
DIP_73	*Chrysomya bezziana*	JQ246660	1164	100%	0.0	100%
OK_30	*Chrysomya nigripes*	OR044072	1205	100%	0.0	100%
OK_31	*Chrysomya nigripes*	OR044072	1099	100%	0.0	100%
OK_33	*Chrysomya pinguis*	KY020777	1098	100%	0.0	100%
OK_32	*Chrysomya pinguis*	KY001888	1199	100%	0.0	100%
OK_34	*Chrysomya pinguis*	KY020777	1081	100%	0.0	100%
OK_40	*Chrysomya defixa*	LC549104	1197	100%	0.0	100%
OK_41	*Chrysomya defixa*	LC549104	1138	100%	0.0	100%
DIP_75	*Hemipyrellia ligurriens*	FJ614821	1107	100%	0.0	100%
DIP_76	*Hemipyrellia ligurriens*	KF562104	1149	99%	0.0	99.68%
DIP_77	*Hemipyrellia ligurriens*	FJ614821	928	100%	0.0	100%
DIP_78	*Hemipyrellia ligurriens*	FJ614821	931	100%	0.0	100%
DIP_90	*Hemipyrellia pulchra*	KR921680	1175	100%	0.0	100%
DIP_91	*Hemipyrellia pulchra*	KR921680	1138	100%	0.0	100%
DIP_79	*Calliphora vomitoria*	KY001919	1195	100%	0.0	100%
DIP_80	*Calliphora vomitoria*	KY001919	1142	100%	0.0	100%
DIP_88	*Calliphora vicina*	KF225212	1164	100%	0.0	100%
DIP_89	*Calliphora vicina*	KF918982	1144	100%	0.0	100%
OK_50	*Calliphora pattoni*	DQ345095	1140	100%	0.0	100%
OK_51	*Calliphora pattoni*	DQ345095	1112	100%	0.0	100%
OK_5	*Lucilia papuensis*	MH540746	1157	99%	0.0	98%
OK_10	*Lucilia papuensis*	MH540746	1116	100%	0.0	98.57%
OK_12	*Lucilia papuensis*	MH540746	1107	100%	0.0	98.72%
OK_11	*Lucilia porphyrina*	KX096343	1225	100%	0.0	99.85%
OK_3	*Lucilia porphyrina*	KX893336	1232	100%	0.0	100%
OK_15	*Lucilia porphyrina*	KX096343	1184	100%	0.0	100%
OK_16	*Lucilia porphyrina*	KX096343	1125	100%	0.0	100%
OK_17	*Lucilia sericata*	KX161623	990	100%	0.0	100%
OK_18	*Lucilia sericata*	KX161623	1164	100%	0.0	100%
OK_19	*Lucilia ampullacea*	MZ608941	1164	100%	0.0	100%
OK_20	*Lucilia ampullacea*	MZ608941	1146	100%	0.0	100%
OK_21	*Lucilia ampullacea*	MZ608941	1136	100%	0.0	100%
OK_22	*Lucilia illustris*	KC510144	1179	100%	0.0	100%
OK_23	*Lucilia illustris*	KJ394905	1109	100%	0.0	100%
OK_42	*Lucilia cuprina*	JN014888	1192	100%	0.0	100%
OK_43	*Lucilia cuprina*	JN014888	1195	100%	0.0	100%

**Table 4:** Identification of 17 collected blow fly species through BOLD identification engine.

Species name	Results of identification (n)	Best identified species	Identity percentage in the BOLD system	BIN number
*Chrysomya rufifacies*	Correct (5)	*C. rufifacies*	99.83%−100%	BOLD:AAB3064
*Chrysomya megacephala*	Correct (8)	*C. megacephala*	99.38%−100%	BOLD:AAA5667
*Chrysomya bezziana*	Correct (4)	*C. bezziana*	100%	BOLD:AAC4787
*Chrysomya nigripes*	Correct (2)	*C. nigripes*	100%	BOLD:AAD0731
*Chrysomya pinguis*	Correct (3)	*C. pinguis*	100%	BOLD:ACF0516
*Chrysomya defixa*	Correct (2)	*C. defixa*	99.84%	BOLD:AAA5667
*Hemipyrellia ligurriens*	Correct (4)	*H. ligurriens*	100%	BOLD:AAE4423
*Hemipyrellia pulchra*	Correct (2)	*H. pulchra*	99.84%	BOLD:AAE4423
*Calliphora vomitoria*	Correct (2)	*C. vomitoria*	100%	BOLD:AAA8931
*Calliphora vicina*	Correct (2)	*C.* (*C.*) *vicina*	100%	BOLD:AAB6579
*Calliphora pattoni*	Correct (2)	*C. (C.) pattoni*	100%	BOLD:AAB6579
*Lucilia papuensis*	Correct (3)	*L. papuensis*	100%	BOLD:ACD6729
*Lucilia porphyrina*	Correct (4)	*L. porphyrina*	99.47%−100%	BOLD:AAC3450
*Lucilia sericata*	Correct (2)	*L. sericata*	100%	BOLD:AAA6618
*Lucilia ampullacea*	Correct (3)	*L. ampullacea*	100%	BOLD:AAC3450
*Lucilia illustris*	Correct (2)	*L. illustris*	100%	BOLD:AAA7470
*Lucilia cuprina*	Correct (2)	*L. cuprina*	100%	BOLD:AAA6618

### Species genetic divergence and phylogenetic analysis

Intraspecific divergences of values < 2% [28] were observed in all species, ranging from 0.00% to 1.00% (Table 5). Intraspecific divergences of 1% were observed in the species *Lucilia papuensis* and *Hemipyrellia pulchra*. All the other species showed 0% intraspecific distance. The range of interspecific divergences was 0.39% to 12.29% (Table 5). The maximum interspecific genetic divergence was seen between *Chrysomya rufifacies* and *Lucilia ampullacea* (12.29%). The least genetic divergences were seen between *Chrysomya defixa* and *Chrysomya megacephala* (0.39%) and *Lucilia sericata* and *Lucilia cuprina* (0.39%). Also, very low interspecific genetic distances were shown by *Calliphora vicina* and *Calliphora pattoni* (0.80%), *Hemipyrellia ligurriens* and *Hemipyrellia pulchra* (1.20%), and *Lucilia porphyrina* and *Lucilia ampullacea* (1.26%). Most of the species of the genus *Chrysomya*, *Lucilia* and *Calliphora* exhibited >2% interspecific genetic divergences among their species.

**Table 5:** The interspecific and intraspecific genetic distances (K2P model) of the 138 COI sequences of calliphorid fly specimens collected throughout West Bengal and the GenBank database.

Species	Intra Specific Distance	1	2	3	4	5	6	7	8	9	10	11	12	13	14	15	16	17	18	19	20	21
1. *C. rufifacies*	0.0																					
2. *C. megacephala*	0.0	6.46																				
3. *C. bezziana*	0.0	7.88	3.33																			
4. *C. albiceps*	0.0	1.69	4.57	5.93																		
5. *C. chani*	0.0	5.14	3.67	4.56	4.19																	
6. *C. nigripes*	0.0	7.00	4.66	5.56	5.10	5.92																
7. *C. pinguis*	0.0	8.27	2.39	4.13	6.30	4.47	5.51															
8. *C. thanomthini*	0.0	6.22	1.41	3.11	4.35	3.45	4.44	03.03														
9. *C. villeneuvi*	0.0	3.37	5.91	7.31	2.88	6.30	7.39	7.70	4.78													
10. *C. defixa*	0.0	6.93	0.39	2.89	5.02	4.11	4.22	2.81	1.83	6.37												
11. *H. ligurriens*	0.0	9.71	8.67	8.67	9.15	8.26	10.24	8.59	8.43	9.62	9.16											
12. *H. pulchra*	1	9.71	8.67	8.67	9.15	8.26	10.24	9.57	7.48	8.65	9.16	1.20										
13. *C. vomitoria*	0.0	6.95	7.18	8.13	5.97	4.12	8.67	8.05	6.95	7.79	7.65	6.72	6.72									
14. *C. vicina*	0.0	8.76	6.37	8.28	6.79	5.88	8.79	8.18	5.23	6.34	6.84	8.72	7.75	3.79								
15. *C. pattoni*	0.0	8.76	6.37	8.28	6.79	5.88	8.60	8.18	5.23	6.34	6.84	9.21	8.23	4.23	0.80							
16. *L. cuprina*	0.0	11.17	8.66	9.64	11.09	7.19	11.19	9.55	8.42	10.60	9.15	6.82	7.29	6.29	6.86	7.34						
17. *L. papuensis*	1	10.11	10.40	11.60	10.04	7.25	12.02	11.32	9.07	8.74	10.91	8.66	7.69	7.16	7.75	7.42	6.34					
18. *L. porphyrina*	0.0	10.68	9.13	11.13	9.61	7.79	10.753	10.07	9.87	10.12	9.62	7.27	6.80	6.26	6.84	7.80	5.47	5.43				
19. *L. sericata*	0.0	10.67	8.18	9.15	10.59	6.73	10.69	9.06	7.95	10.10	8.66	6.35	6.82	5.83	6.39	6.86	0.39	5.87	5.02			
20. *L. ampullacea*	0.0	12.29	10.19	12.25	11.18	8.81	11.50	11.16	9.94	10.69	10.69	8.30	6.87	7.72	7.38	7.38	6.93	5.19	1.26	6.46		
21. *L. illustris*	0.0	9.21	9.61	10.10	8.18	8.11	11.19	10.52	8.41	6.79	10.10	5.93	5.47	5.51	5.00	5.44	4.14	4.55	3.72	3.70	4.22	

**Fig 3:**
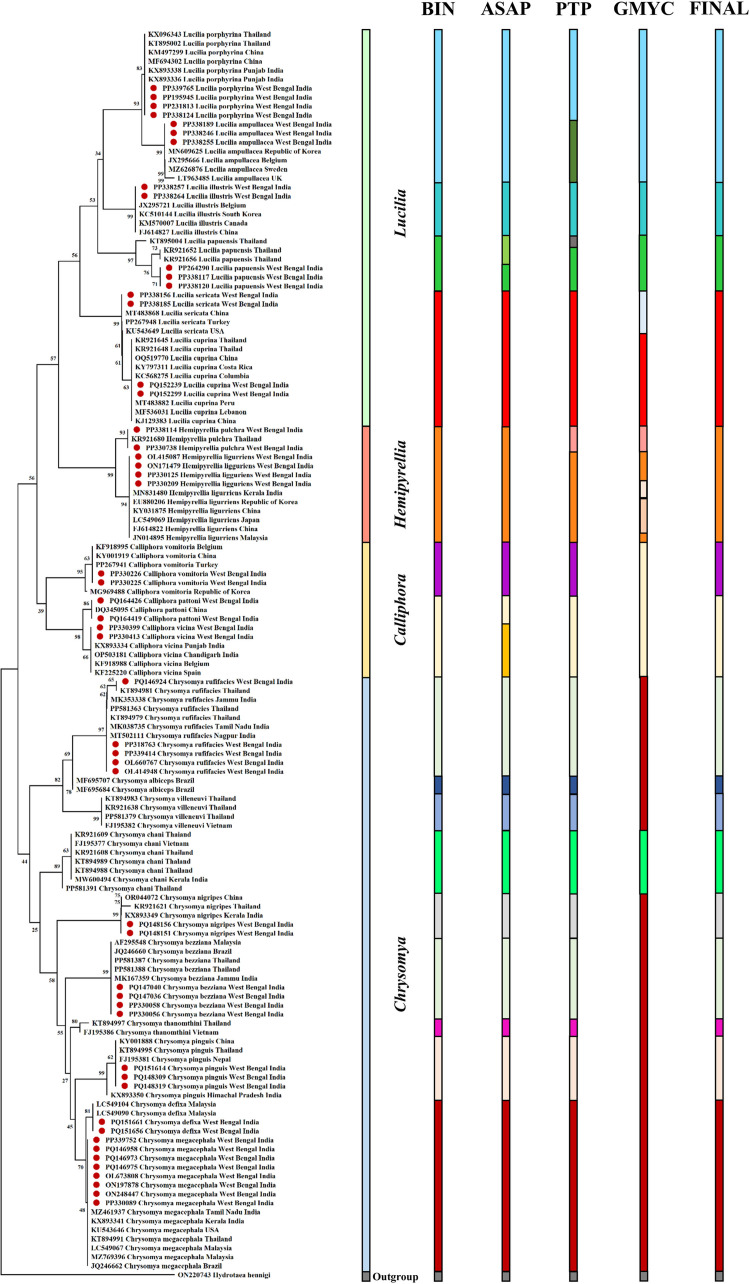
Neighbour-joining (NJ) tree of 21 calliphorid species with bootstrap support values with the comparison of different species delimitation methods. Barcode Index Number (BIN) system, Assemble Species by Automatic Partitioning (ASAP), Poisson Tree Processes (PTP), Generalised Mixed Yule Coalescent (GMYC), and FINAL (Final identification).

Neighbour-Joining (NJ), Maximum Likelihood (ML) and Bayesian (BA) analyses recovered the reciprocal monophyly of the morphologically studied calliphorid species, with significant branch support for all methods. The Neighbour Joining (NJ) tree, Maximum-Likelihood (ML), and Bayesian (BA) tree effectively distinguished the twenty-one blowfly species based on monophyletic separation except that *Chrysomya megacephala* and *Chrysomya defixa* showed paraphyly in both Maximum Likelihood (ML) and Bayesian (BA) trees. At the species level, the high bootstrap values in deep branches for NJ and ML trees suggested support for monophyly. All trees recovered the monophyly of the four genera, *Chyrsomya*, *Calliphora*, *Hemipyrellia*, and *Lucilia* with high support values. All COI sequences from this study clustered with respective fly species previously examined from other parts of the world and India (Table 1) with strong bootstrap values in most of the branches in NJ tree (Fig 3). Maximum likelihood and Bayesian analysis yielded similar results showing well resolved trees with strong bootstrap and posterior probability support for most of the branches, recovering the monophyly of all species except *C. megacephala* that was paraphyletic (S1 and S2 Figs).

Additional haplotype analysis revealed that blow fly species, including those generated from GenBank and ours, had 30 haplotypes, showing moderate to high haplotype diversity (0.953) (Table 6). The COI gene revealed that *Lucilia papuensis* had the highest number of haplotypes (3) along with the highest haplotype and nucleotide diversity values of 0.73 and 0.00870, respectively. The segregating sites for *Lucila papuensis* were the highest, with a value of 5.

**Table 6:** Haplotype diversity and number of mitochondrial haplotypes.

Name of specimens	Number of sequences	Average number of differences (K)	Number of haplotypes	Haplotype Diversity (H_d_)	Nucleotide Diversity (P_i_)	Number of segregating sites (S)
*Chrysomya rufifacies*	11	0.327	2	0.327	0.00129	1
*Chrysomya megacephala*	15	0.00	1	0.00	0.00	0
*Chrysomya bezziana*	9	0.00	1	0.00	0.00	0
*Chrysomya albiceps*	2	0.00	1	0.00	0.00	0
*Chrysomya chani*	7	0.285	2	0.285	0.00113	1
*Chrysomya nigripes*	5	0.400	2	0.400	0.00158	1
*Chrysomya pinguis*	7	0.285	2	0.285	0.00113	1
*Chrysomya thanomthini*	2	1.00	2	1.00	0.00395	1
*Chrysomya villeneuvi*	4	0.00	1	0.00	0.00	0
*Chrysomya defixa*	4	0.00	1	0.00	0.00	0
*Hemipyrellia ligurriens*	10	0.00	1	0.00	0.00	0
*Hemipyrellia pulchra*	3	0.00	1	0.00	0.00	0
*Calliphora vomitoria*	6	0.333	2	0.333	0.00132	1
*Calliphora vicina*	6	0.00	1	0.00	0.00	0
*Calliphora pattoni*	3	0.00	1	0.00	0.00	0
*Lucilia cuprina*	10	0.00	1	0.00	0.00	0
*Lucilia papuensis*	6	2.20	3	0.733	0.00870	5
*Lucilia porphyrina*	10	0.00	1	0.00	0.00	0
*Lucilia sericata*	5	0.00	1	0.00	0.00	0
*Lucilia ampullacea*	7	0.285	2	0.285	0.00113	1
*Lucilia illustris*	6	0.00	1	0.00	0.00	0

### Species delimitation analysis

The four delimitation methods, BIN, ASAP, PTP, and GMYC, used for species identification, produced similar results (Fig 3). MOTUs without a match were not found. Out of the 10 “best” partitions determined by the ASAP analysis, the fourth partition ranked first, selected with an ASAP score of 4.5 and a threshold distance of 0.0099. ASAP analysis formed 18 MOTUs. ASAP analysis was comparable with BIN and single PTP analysis, which produced 16 and 19 MOTUs, respectively. 14 MOTUs matched between ASAP and BIN analyses, while 12 MOTUs matched between ASAP and PTP analyses. GMYC analysis produced 13 MOTUs, which was less in number. Three analyses, including ASAP, single PTP, and BIN, showed good agreement and congruency, although GMYC revealed less numbers of putative species. *Chrysomya megacephala* and *Chrysomya defixa*, *Calliphora vicina* and *Calliphora pattoni*, *Lucilia porphyrina* and *Lucilia ampullacea*, and *Lucilia sericata* and *Lucilia cuprina* were considered as one species group by most of the delimitation methods. However, each showed monophyly in the NJ tree. *Hemipyrellia ligurriens* and *Hemipyrellia pulchra* were regarded as one by BIN and ASAP methods, whereas PTP and GMYC considered them separate species. *Calliphora vicina* and *Calliphora pattoni* were considered separate species in the ASAP method. *Lucilia porphyrina* and *Lucilia ampullacea* were considered separate species in the PTP method. *Lucilia papuensis* of Thailand (KT895004) was separated from the species cluster in PTP analysis. Despite varying results, ASAP, GMYC, and PTP methods were fairly congruent with the BIN method.

## Discussion

The findings of this DNA barcoding investigation provide crucial light on the distribution and variety of forensically relevant fly species in four different geo-climatic zones of West Bengal. The discovery of these forensically relevant calliphorid species demonstrates the diverse ecological niches and habitat preferences of these flies, which are impacted by various environmental factors. *Chrysomya megacephala* was the most abundant and dominant species across all four geo-climatic regions of West Bengal, especially in Gangetic plains, coastal areas, and arid regions, indicating its importance as a primary colonizer of decomposing carcasses. The warm and humid climatic conditions with the quick arrival of decaying remains make it one of the essential forensic indicators along with sarcophagid and muscid species [2,66,67]. The species second to *C. megacephala* in abundance was *Chrysomya rufifacies*, which was similarly abundant in the Gangetic plains, arid regions, and coastal areas. Its preference for climatic conditions was similar to *C. megacephala*. Other *Chrysomya* species like *C. bezziana*, *C. pinguis*, *C. nigripes*, and *C. defixa* were fairly abundant in the warmer and humid regions, indicating their preference for such climatic conditions. *Hemipyrellia ligurriens* and *Hemipyrellia pulchra* showed moderate abundance and equal distribution across all four geo-climatic regions, demonstrating their broad ecological range. Their numbers were high during the monsoon season, indicating their preference for moist conditions. They generally visit dead remains in the later decomposition stages. *Calliphora vomitoria*, *Calliphora vicina*, and *Calliphora pattoni* were generally restricted to the hilly regions during the pre-monsoon and monsoon seasons. Their absence in the warmer climatic conditions is indicative of a preference for cooler regions. Being active in colder areas makes them useful for forensic work, as their appearance can point to higher elevation death incidents or bodies that have been moved from those places. *Lucilia illustris*, *Lucilia papuensis*, *Lucilia porphyrina*, and *Lucilia sericata* were more or less abundant and uniformly distributed in the Gangetic plains and coastal areas, indicating their preference for warm and humid temperatures. *Lucilia cuprina* was abundant in the coastal regions. *Lucilia ampullacea* was predominantly found in hilly regions, along with the presence of other *Lucilia* species. In the higher altitudes, there was an even distribution of other *Lucilia* species except *Lucilia illustris*, which was completely absent.

The monsoon months had greater species abundance and diversity in most environments. This is because it has the best temperature and humidity levels for oviposition and larval development. Post-monsoon activity, on the other hand, dropped down overall, possibly because of less moisture and cooler temperatures. On the other hand, pre-monsoon activity was high early in the season, notably in Gangetic plains, arid, and coastal areas. These results show how important it is for forensic studies to have entomological baselines specific to the area. When estimating the post-mortem interval, it is important to take into account how the species diversity and abundance change depending on the environment. Overall, the results make it clear that localised blow fly data is needed to improve the accuracy of forensics in a variety of environments.

The molecular identification of 98%−100% similarity match from the NCBI and BOLD databases has increased the accuracy of identifying these blow fly species [57]. In [28] it is stated that the capacity of DNA-based species identification techniques to distinguish between intraspecific and interspecific differences is crucial. To ensure the reliability of the gene used for species-level identification, the interspecific and intraspecific genetic divergence for any recently diverged species should be 2% [28,89]. There was no overlap between intraspecific and interspecific distances in this study, which indicated that all the studied species were properly identified. In our studied dataset, *H. ligurriens* and *H. pulchra*, *Chrysomya defixa* and *Chrysomya megacephala*, *L. sericata* and *L. cuprina*, *L. porphyrina* and *L. ampullacea*, and *Calliphora vicina* and *Calliphora pattoni*, despite their apparent morphological differences, are sister species as depicted by all the topologies. But their interspecific genetic distances were lower than 2%, and the species delimitation results grouped them as one species. This indicates that they have diverged recently, and more genetic markers will improve the delimitation results. Substantial barcode gaps between the different genera and remaining species showed that they differed because of enough COI genetic divergences. Unlike other arthropod species, insects generally have less genetic variation and intraspecific divergence in all animal taxa, hardly exceeding 2% [28]. The large barcoding gap (i.e., 0%−1% for intraspecific and 0.39%−12.29% for interspecific divergence) seen in the present study implies that all species examined here, except the aforementioned species exhibit sufficient COI sequence divergencies for their accurate species identification. Another mitochondrial or nuclear marker will be suitable for more accurate differentiation of these sister species. A common and traditional approach for the barcoding methodology is the barcode gap based on K2P distances and ML analysis, although it is unsuitable for species delineation [90,91].

In delimitation analyses, congruency was seen between ASAP, BIN, GMYC, and single PTP as it was seen in studies on molecular identification of forensically significant dipterans [55,57]. *C. megacephala* and *C. defixa*, *C. vicina* and *C. pattoni*, *L. porphyrina* and *L. ampullacea*, and *L. sericata* and *L. cuprina* pairs were grouped together as single-species. Although being sister species and monophyly shown in the NJ method, more molecular marker other than COI might have potential to delimit them as separate species. As suggested by [24,92] we employed the three most popular phylogenetic tree-building techniques to analyze our data and ensure that the final tree is flexible for the many underlying presumptions made by each method. NJ, ML, and BI analyses restore the reciprocal monophyly of the COI sequences generated from the previously morphologically studied species, with significant branch support for all methods. A significant paraphyly between *C. megacephala* and *C. defixa* was observed in the ML and BI analyses, which was a bit similar to the work of [39], where there was paraphyly shown between *C. megacephala* and *C. saffranea*. However, in the NJ method, no paraphyly was observed between *C. megacephala* and *C. defixa*. The constructed NJ tree of COI shared similarities with the studies of forensically significant blow flies from Thailand [52], in which it was shown that 16 species of calliphorids had high bootstrap support. Also, the NJ tree showed similar monophyly as seen in the DNA-based identification of forensically important sarcophagids from Australia [93]. All branches showed strong monophyly, and *Chrysomya*, *Lucilia*, and *Hemipyrellia* genera were distinctly separated. The ML tree and BI tree showed congruency with the works of [24]. The two genera, namely *Chrysomya* and *Callihpora*, showed strong monophyly, and the *Lucilia* genus showed weakly supported monophyly. The ML tree showed a similar topology to the works of [39,51], where the *Chrysomya* sister group to the *Calliphora*-*Lucilia* clade. Closely related species *Chrysomya megacephala* and *Chrysomya bezziana* showed strong monophyly. Both species look similar and are differentiated based on very intricate taxonomic characters. The same goes for *Hemipyrellia pulchra* and *Hemipyrellia ligguriens*, where both showed strong monophyly. Both species look similar and are often confused due to the variable color of the antennae. In the *Lucilia* and *Calliphora* genera, it is also seen that all the species are very closely related and differentiate through intricate characters and genitalia. All these results are indicative of an integrative approach for quick and proper identification of these forensically important blow flies from this region of India. It will aid the forensic experts in minPMI estimation of forensic investigations involving both criminal and animal poaching cases. In conclusion, this study demonstrated that the accuracy of species identification was produced by both the morphological identification of flies and nucleotide sequences utilizing the DNA barcoding technique. Also, the information about the distribution and diversity of these flies from the four different geo-climatic regions will expedite the process of determination of corpse relocation in forensic investigations and crime cases.

## Conclusions

Identifying and distinguishing the medically and forensically significant calliphorid flies is essential to comprehend and regulate their impacts in epidemiology, medicine, forensics, and veterinary. The COI gene shows potential as a DNA-based identification approach for Indian blow flies, especially from immature larval or damaged specimens. There was a 100% similarity value between the molecular and morphological attributes. They accurately identify fly species that are important for forensic purposes. Forensic investigations will be significantly aided by the ease with which DNA barcoding techniques may identify these fly samples. This approach bypasses the limitations of conventional taxonomy for calliphorid species and aids in identifying species complexes, including cryptic and visually similar species. More significantly, our study emphasizes how crucial it is to create a comprehensive barcode database for Calliphoridae species in GenBank from this area, as they provide helpful forensic markers for situations involving the illegal hunting of wildlife and criminal investigations.

Accurately identifying blow flies is essential, and it will have a significant impact on medical, epidemiologic, and synanthropic studies in West Bengal and throughout India because of their possible involvement as vectors of various lethal pathogens that cause diseases. Multiple studies have been conducted in northern, central, and southern India, except eastern India. This study is a breakthrough initiative in Eastern India, offering thorough coverage of several geo-climatic zones in West Bengal, including the Gangetic Plains, the Sagar Islands, the arid Chotanagpur plateau region, and the Central Himalayan landscape. Also, COI gene sequences from other parts of India have been included in the study for better understanding. Further, our results emphasize the necessity of adding other genetic (nuclear and mitochondrial) markers to improve the development of precise and robust phylogenetic analyses of blowfly species and genera. Finally, our insights will boost forensic entomological studies in India.

## Supporting information

S1 Fig The ML tree for the blow fly species. (TIF)

S2 Fig The BA tree for the blow fly species. (TIF)
