# Supplementary figures and images for: Molecular identification and genetic variations of forensically significant blow flies (Diptera: Calliphoridae) from Eastern India using DNA barcoding
Source: PLoS One. 2025 Jul 22;20(7):e0327039. doi: 10.1371/journal.pone.0327039 (PMC12282894; doi:10.1371/journal.pone.0327039)

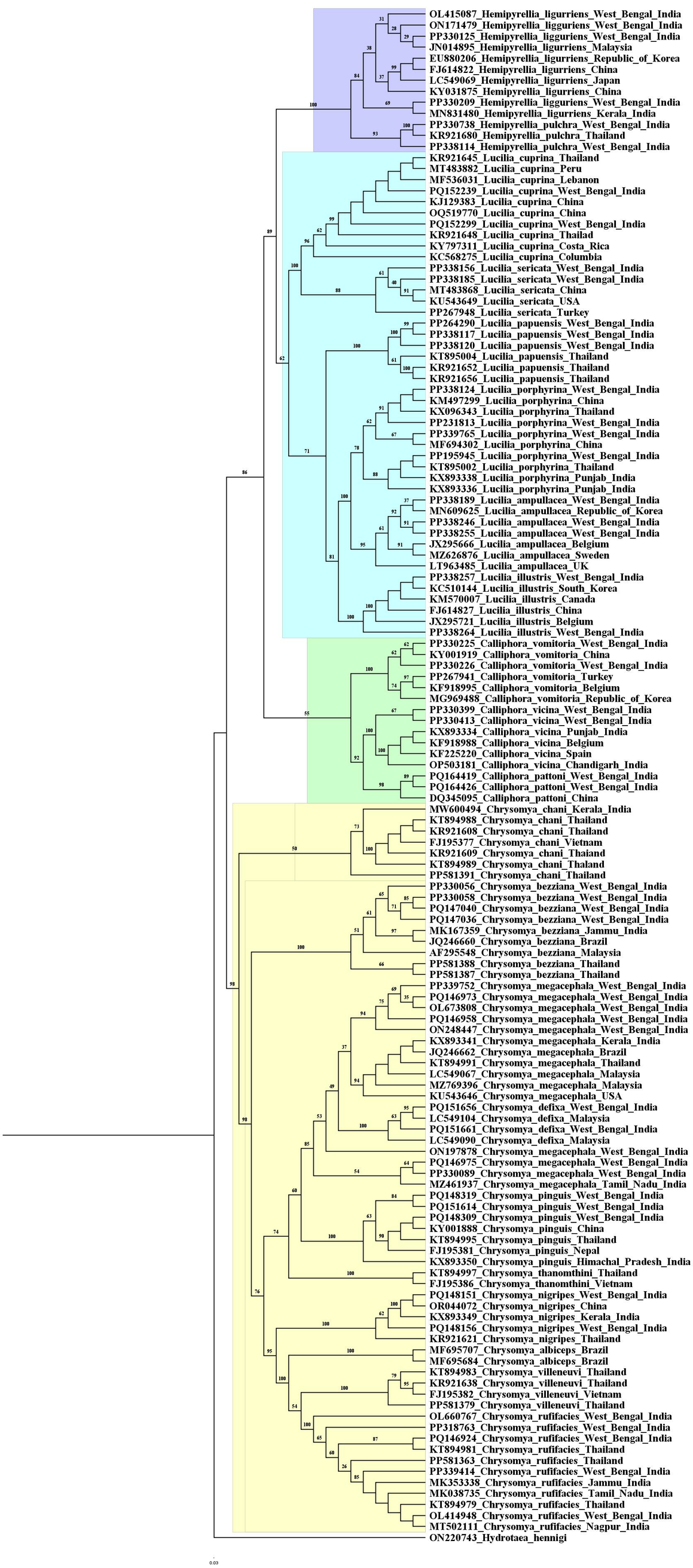

Supplement: S1 Fig — (TIF) [file pone.0327039.s001.tif]

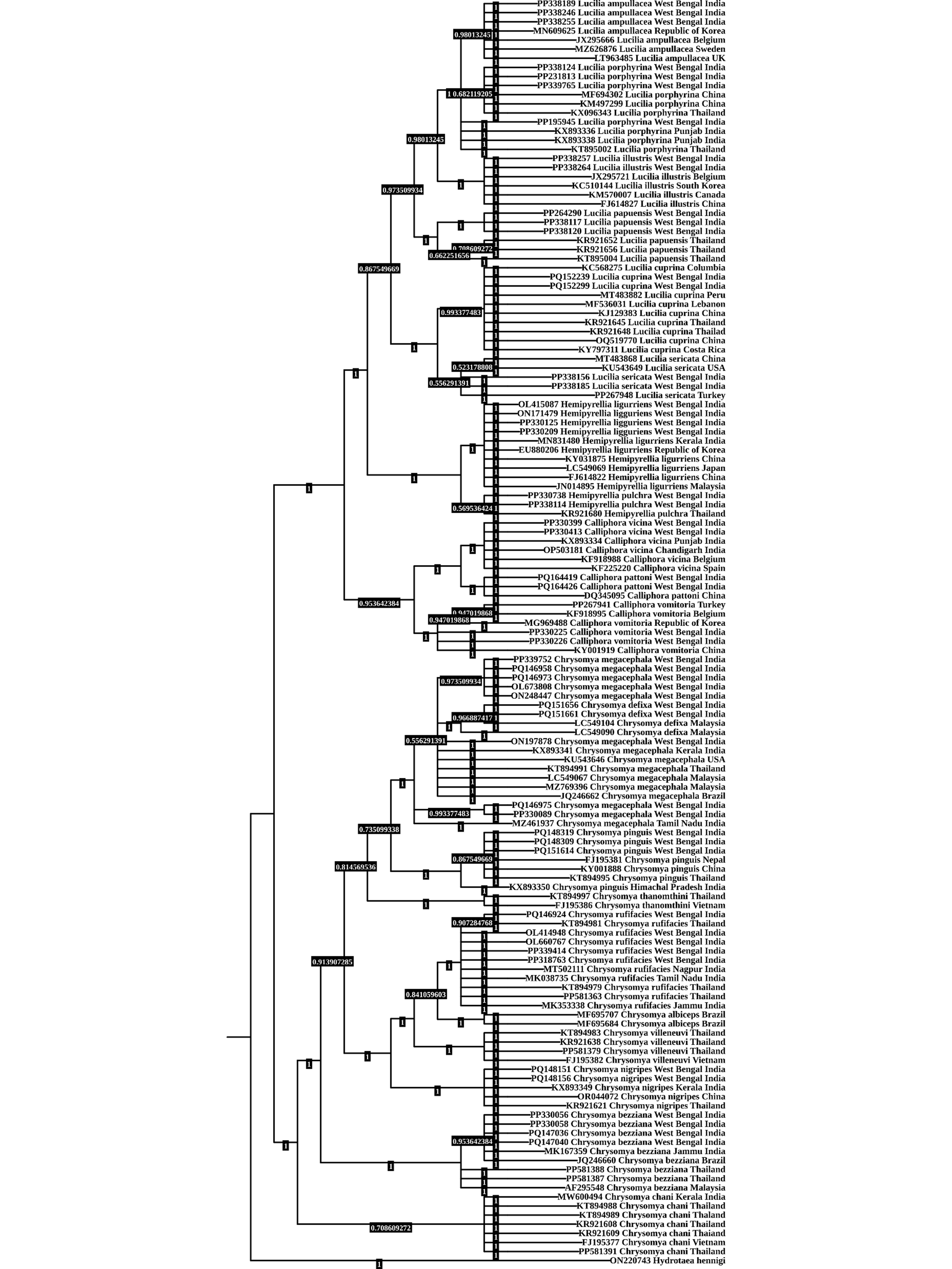

Supplement: S2 Fig — (TIF) [file pone.0327039.s002.tif]
